# A Consulting Institution

**Published:** 1898-12-31

**Authors:** 


### A CONSULTING INSTITUTION.

The proposal to establish in Birmingham a sort of cheap consultation shop for the advantage of various people who wish to have the advice of a "consultant," and yet do not want to pay the ordinary consultation fee, seems to us to be one which, if it should come to anything, would be very injurious to the profession, and one, moreover, which would be in direct contravention of all that is accepted as the correct attitude towards medical aid associations. It matters little whether or not the proposed "Consulting Institution" pays a dividend or not. The medical man who accepted a salary for giving such consultations as are proposed to be given, and allowed his employers to farm out his services for payment, would undoubtedly do exactly the very thing against which the profession and the General Medical Council have united in protesting in regard to medical aid associations. We fancy, therefore, that the suggestion will come to nothing; for, unless physicians connected with hospitals allow themselves to become attached to this institution, it must die out. We feel bound, however, to remark that the medical profession is to a certain extent itself responsible for the constantly recurring attempts, of which this institution is the latest example, to "get at" it, to organise it, and to exploit it for the benefit of outsiders.

